# Prospects of Using Gum Arabic Silver Nanoparticles in Toothpaste to Prevent Dental Caries

**DOI:** 10.3390/pharmaceutics15030871

**Published:** 2023-03-08

**Authors:** Omnia Abdelmoneim Khidir Ahmed, Nicole Remaliah Samantha Sibuyi, Adewale Oluwaseun Fadaka, Ernest Maboza, Annette Olivier, Abram Madimabe Madiehe, Mervin Meyer, Greta Geerts

**Affiliations:** 1 Department of Restorative Dentistry, University of the Western Cape, Bellville 7535, South Africa; 2 Department of Science and Innovation (DSI)/Mintek Nanotechnology Innovation Centre (NIC) Biolabels Research Node, Department of Biotechnology, University of the Western Cape, Bellville 7535, South Africa; 3 Oral and Dental Research Laboratory, University of the Western Cape, Bellville 7535, South Africa

**Keywords:** green synthesis, gum arabic, silver nanoparticles, toothpaste, antimicrobial, cytotoxicity

## Abstract

There is growing interest in the use of green synthesized silver nanoparticles (AgNPs) to control and prevent dental diseases. The incorporation of green synthesized AgNPs into dentifrices to reduce pathogenic oral microbes is motivated by their presumed biocompatibility and broad-spectrum antimicrobial activity. In the present study, gum arabic AgNPs (GA-AgNPs) were formulated into a toothpaste (TP) using a commercial TP at a non-active concentration, to produce GA-AgNPs_TP. The TP was selected after evaluating the antimicrobial activity of four commercial TPs 1-4 on selected oral microbes using agar disc diffusion and microdilution assays. The less active TP-1 was then used in the formulation of GA-AgNPs_TP-1; thereafter, the antimicrobial activity of GA-AgNPs_0.4g was compared to GA-AgNPs_TP-1. The cytotoxicity of GA-AgNPs_0.4g and GA-AgNPs_TP-1 was also assessed on the buccal mucosa fibroblast (BMF) cells using the MTT assay. The study demonstrated that antimicrobial activity of GA-AgNPs_0.4g was retained after being combined with a sub-lethal or inactive concentration of TP-1. The non-selective antimicrobial activity and cytotoxicity of both GA-AgNPs_0.4g and GA-AgNPs_TP-1 was demonstrated to be time and concentration dependent. These activities were instant, reducing microbial and BMF cell growth in less than one hour of exposure. However, the use of dentifrice commonly takes 2 min and rinsed off thereafter, which could prevent damage to the oral mucosa. Although, GA-AgNPs_TP-1 has a good prospect as a TP or oral healthcare product, more studies are required to further improve the biocompatibility of this formulation.

## 1. Introduction

The oral cavity is the second most complex microbiota in the human body after the colon and contains a diverse microbial community [1]. An imbalance in the microbial composition, which can be caused by certain local factors including the consumption of carbohydrates, plaque accumulation, pathogenic oral microbes, and poor oral hygiene, can lead to the development of oral diseases such as dental caries and periodontitis [2,3]. Preventing these diseases through regular brushing and the use of antimicrobial agents added to oral hygiene products assists in the removal of pathogenic microbes from the enamel surfaces and presents a simpler and more economical approach than a later treatment [4].

The use of oral care products such as TPs and mouth rinse is a fundamental means for the control and prevention of oral diseases [5]. The majority of the TPs are composed of three major chemicals, i.e., fluoride (an antimicrobial and dental enamel remineralizing agent), triclosan (an antibacterial agent), and sodium lauryl sulfate (an anionic surfactant with detergent action) [6,7]. In addition, there are other components such as humectants, abrasive agents, and flavoring agents [5]. Although there are many TP formulations with antimicrobial activities, unfortunately, the emergence of antimicrobial-resistant oral microbes necessitates the development of alternative antimicrobial agents that are safe and relatively economical [8]. There is a growing interest in the use of natural products to combat microbial infections [9]. Studies have shown that natural products can be used as reducing agents in the synthesis of metal nanoparticles (MNPs) with enhanced antimicrobial activities [10]. The incorporation of these green synthesized NPs in oral hygiene products was demonstrated to improve their efficacy and expand their field of application. Nanomaterials, or NPs, are defined as materials with one or more dimensions at a size range between 1 and 100 nm and exhibit different properties as compared to their precursors or bulk materials [11,12]. The morphology and size distribution of the NPs are key factors for their inventive bioapplications [13,14,15], including in oral care products [16,17,18,19].

AgNPs are among the MNPs that have received special attention in recent years, especially in biomedicine, due to their immense antibacterial, anti-inflammatory, antiviral, and antifungal activities [13,17,18,20,21,22,23]. They have been synthesized from synthetic and natural compounds, although the biosynthesis route is preferred to reduce the toxicity associated with the chemical synthesis method [10,24]. The synthesis of bioinspired AgNPs involves using microbial (bacteria, fungi) and plant extracts as reducing and stabilizing agents [10,25,26]. Plants have many advantages over microorganisms, as they are economical and easy to process [27]. Bioactive AgNPs have been synthesized using various plant extracts from *Punica granatum* [28], *Justica glauca* leaf [29], *Plectranthus ambionicus* [30], Rice [31], and Gum Arabic [18,32], and have been shown to have broad-spectrum antimicrobial activity against various oral microbes including *Streptococcus mutans* (*S. mutans*), *Candida albicans* (*C. albicans*), *Lactobacillus acidophilus* (*L. acidophilus*), and *Enterococcus faecalis* (*E. faecalis*). Their antimicrobial activity against Gram-positive, Gram-negative bacteria, as well as drug resistant microbes [33], suggests that AgNPs can potentially be used as antimicrobial agents. The bioactivity of AgNPs is influenced by several factors, including, among others, their size, shape, concentration, and surface composition [34,35,36,37].

Due to their potent antimicrobial activity, AgNPs have been incorporated into TPs, toothbrushes, and mouthwashes to combat cariogenic bacteria [38]. AgNPs in dentifrices demonstrated superior activity in reducing the oral microbes associated with dental caries and periodontal diseases [39], while 1.5–2 g of TP is required to inhibit the growth of oral cavity microbes [40]. The antimicrobial activity of a commercial nanosilver-containing TP (TruCare Nano Silver TP) against *S*. *mutans* was compared to fluoride-based TP (Oral B Pro Health) and chitosan TP (Conybio Plus Chitosan Dental TP) and showed favorable and superior effects [4]. Previously, a commercial TP (Royal denta) containing AgNPs (R.D. Silver) was reported to be most effective against *Staphylococcus aureus* (*S. aureus*) and *E. faecalis*, while neither R.D. Silver nor “Royal denta” TP containing gold nanoparticles as well as tangerine and orange oils (R.D. Orange and Gold) had effect on *Escherichia coli* (*E. coli*) or *Proteus mirabilis* (*P. mirabilis*) [40].

The bioactivity of AgNPs has been hampered by contradictory reports regarding their toxic effects to human cells and warrants further studies on their mechanism of action as it is still not clear. Therefore, it is imperative to know their adverse effects in humans and to understand how they behave within biological systems before their clinical application [41]. It was indicated that AgNPs, after oral exposure, could be responsible for inflammation of the gastrointestinal tract, weight loss, disruption of blood biochemistry, and dysfunction of liver enzymes [42]. In an in vitro study by Tang et al., AgNPs were toxic to human gingival epithelial cells and significantly reduced cell viability at concentrations ≥20 µg/mL [43]. Since TPs have been suggested for topical application and not for systematic use, it is unlikely that harmful effects would be observed [41]. The aim of the present study was to evaluate whether the antimicrobial activity of green synthesized GA-AgNPs can be altered when incorporated into a TP formulation.

## 2. Materials and Methods

### 2.1. Antimicrobial Activity of the Commercial TPs

#### 2.1.1. Preparation of the Commercial TPs

Four commercial TPs (TP1-4) were purchased from a local pharmacy in Cape Town (South Africa). Three of the TPs (TP 1-3) were fluoride-based TPs, and one (TP-4), was a charcoal-based TP. The TPs were prepared as a slurry following a previous protocol [44], where 5 g of the TPs was dissolved in 10 mL of Mueller-Hinton Broth (MHB; Sigma Aldrich, St Louis, MO, USA) and vortexed until completely resuspended in solution. The concentration of each TP in solution was 500 µg/mL and considered as 100%.

#### 2.1.2. Microorganisms and Culture Conditions

The antimicrobial activity of the TP1-4 was investigated by agar disc diffusion and microdilution assays in one fungal and three bacterial strains: *Streptococcus sanguinis* - *S. sanguinis* (NCTC 7865; Davies Diagnostics, Randburg, South Africa), *S. mutans* (NCTC 10449; Davies Diagnostics), *L. acidophilus* (ATCC 314; American Type Culture Collection (ATCC) Manassas, VA, USA) and *C. albicans* (ATCC 10231). A single colony was sub-cultured in Brain Heart Infusion Agar (Sigma Aldrich) for all the bacterial strains and Sabouraud dextrose agar for *C*. *albicans* at 37 °C for 24 h. Following the overnight culture, the microbes were adjusted to 0.5 McFarland (McF) standard using DensiCHEK Plus (BioMérieux Inc., Durham, NC, USA).

##### Agar Disc Diffusion Assay

The agar disc diffusion method was used to determine the antimicrobial activity of the commercial TP 1-4 on *S. sanguinis*, *S. mutans*, *L. acidophilus* and *C. albicans*, as described before [18]. In this method, agar plates were inoculated with a 100 µL of each of the standardized inoculum of the test microorganisms. Filter paper discs (6 mm in diameter) previously infused with 100 µL of each of the four commercial TPs (stock concentration of 500 µg/mL) and air-dried overnight were placed on the inoculated agar plates. Then, 0.2% chlorohexidine (CHX) and 5000 u Nystatin were used as positive controls for bacteria and fungi, respectively, and water was used as a negative control. The plates were incubated for 24 h at 37 °C and observed for zones of inhibition (ZOI) by measuring the diameters of ZOI formed around the filter paper disk using a Vernier caliper. The assay was carried out in triplicate for all tested organisms and repeated six times.

##### Microdilution Assay

The commercial TP with the least antimicrobial activity from the disc diffusion assay was used in this experiment. The lowest concentration of the commercial TP that visually inhibited growth of the four microorganisms (Minimum Inhibitory Concentrations, MICs) was determined according to the protocol set by the M07 of the Clinical Laboratory Standards Institute (CLSI) [45].

The 0.5 McF of the test microbes was added in 96-well plates (100 µL/well) and incubated at 37 °C for 24 h. The plates were rinsed with phosphate buffered saline (PBS; Sigma Aldrich) three times and 100 µL of MHB was pipetted into all wells. Then, 100 µL of TP (3.9–500 µg/mL) was transferred into the wells, except for the untreated controls, where 100 µL of MHB was added. The plates were incubated at 37 °C for 24 h. Then, the MICs were determined by sub-culturing 5 µL of samples from each well on a Tryptic Soy Agar plates (TSA; Sigma Aldrich) and incubating at 37 °C for 24 h following a previous method [46]. The growth or inhibition of *S. mutans* was qualitatively and quantitatively determined by visually examining the culture media and counting the total number of colonies formed (colony forming unit, CFU) on TSA to determine MICs. The experiment was carried out in triplicate and repeated three times.

### 2.2. Synthesis of GA-AgNPs and Preparation of the GA-AgNPs_TP

#### 2.2.1. Synthesis and Characterization of GA-AgNPs

The GA-AgNPs_0.4g used in this study were synthesized and characterized as previously described [18]. Briefly, 1.6 g of *Acacia senegal* gum arabic (GA) powder purchased from local vendors in North Kordofan (Sudan, Africa) was dissolved in 100 mL of boiling deionized water and filtered through 0.45 µm filters before use. Then, 4 g of silver nitrate (AgNO_3_; Sigma Aldrich) dissolved in 100 mL of water was added to the GA solution. The volume of GA/AgNO_3_ solution was adjusted to 400 mL with deionized water, quickly mixed by swirling the flask and autoclaved at 120 °C at 15 psi pressure for 20 min. The GA-AgNPs_0.4g were centrifuged at 9000 rpm for 45 min, resuspended in equal volume of deionized water then stored at room temperature covered in foil until further analysis.

Characterization of the GA-AgNPs_0.4g was previously reported. Briefly, the AgNPs were analyzed by ultraviolet-visible (UV-Vis) spectrophotometer on a POLARstar Omega plate reader (BMG LABTECH, Offenburg, Germany), dynamic light scattering (DLS) using Malvern NanoZS90 Zetasizer (Malvern Panalytical Ltd., Enigma Business Park, UK), Transmission Electron Microscopy (TEM) at the Electron Microscope Unit (University of Cape Town, South Africa) by a TecnaiF20 HRTEM (FEI Company, Hillsboro, OR, USA), and Fourier-Transform Infrared Spectroscopy (FTIR) at UWC School of Pharmacy using Perkin Elmer Spectrum Two FTIR spectrophotometer (Waltham, MA, USA) [18].

#### 2.2.2. Preparation of the GA-AgNPs_TP-1

GA-AgNPs_TP-1 stock was prepared fresh by mixing 5 mL of TP-1 slurry (62.5 µg/mL) and 5 mL of 200 µg/mL GA-AgNPs_0.4g. The mixture was vortexed for 5 min. The concentration of TP-1 was kept constant at 62.5 µg/mL and used for further serial dilutions of GA-AgNPs_0.4g to yield 6.25–100 µg/mL (final concentration of TP-1 in GA-AgNPs_TP-1 treatments was 31.3 µg/mL).

### 2.3. Antimicrobial Activity of the GA-AgNPs-TP

#### 2.3.1. Agar Disc Diffusion Method

The antimicrobial activity of the GA-AgNPs_0.4g and the GA-AgNPs_TP-1 was compared using the agar disc diffusion method, as described in Section 2.1.2, on the four microorganisms. Filter paper discs were infused with 100 µL of each of the two treatments at varying concentrations from 6.25 to 100 µg/mL and allowed to air-dry overnight. After spreading 100 µL (0.5 McF) of each inoculum on a sterile agar plate, the discs were placed on the agar surface. Then, 0.2% CHX and 5000u Nystatin were used as positive controls for bacteria and fungi, respectively, and the 62.5 µg/mL TP-1 was also used as a control. The plates were incubated for 24 h at 37 °C, then the plates were observed for ZOI and the diameters of ZOI were measured using a Vernier caliper. The assay was carried out in triplicate for all the tested microorganisms and repeated three times.

#### 2.3.2. Microdilution Assay

The MICs for GA-AgNPs_0.4g and the GA-AgNPs_TP-1 were determined by broth microdilution assay as described above. Briefly, serially diluted concentrations of GA-AgNPs_0.4g and GA-AgNPs_TP-1 were prepared in MHB ranging from 6.25 to 100 µg/mL. MHB medium containing the standard inoculum was added to the serially diluted concentrations of the treatment. After 24 h of incubation at 37 °C, the wells were checked for any evidence of microbial growth by sub-culturing a sample from each well on TSA and incubated at 37 °C for 24 h. The growth or inhibition of the microbes was determined by visually examining the culture media followed by counting the total number of colonies formed (CFU) on TSA to determine MICs for the treatments.

### 2.4. Cytotoxicity Assay

Two experiments were performed in this assay. In the first experiment, the MTT (3-[4,5-dimethylthiazol-2-yl]-2,5 diphenyl tetrazolium bromide; Sigma Aldrich) assay was used to assess the cytotoxicity of TP-1, GA-AgNPs_0.4g and the GA-AgNPs_TP-1 on BMF cells, following a previous method [47]. The BMF cells is a Human Oral Fibroblast cell line established in the Oral and Dental Research Institute (UWC, South Africa). The cells were maintained and grown to near confluence in Dulbecco’s Modified Eagle’s Medium (DMEM) supplemented with 10% fetal bovine serum (Hyclone laboratories, South Logan, UT, USA) and 1% penicillin-streptomycin cocktail (Gibco, Germany). The cells were seeded in a 96 well plate (100 µL/well) at 2 × 10^4^ cells per well and allowed to grow for 24 h in a water jacketed CO_2_ incubator (Forma series 3-model 4111, Thermo scientific). The cells were treated with 100 µL of the treatments ranging from 6.25 to 100 µg/mL. The TP-1, GA-AgNPs_0.4g, and GA-AgNPs_TP-1 treatments were prepared in DMEM. After 24 h, the cell viability was assessed by adding 10 µL of 5mg/mL MTT solution to each well and incubated at 37 °C for 3 h. Then, 100 μL of DMSO (Sigma Aldrich) was added to all wells and the absorbance was measured at 570 nm using an RT-2100C microplate reader (Rayto Life and Analytical Sciences Co., Shanghai, China). The percentage of cell viability was calculated using the following equation:

% Cell Viability = (sample absorbance/control absorbance) × 100%

In the second experiment, the time point and concentration of the treatments where the cytotoxicity on the BMF cells started was investigated. The same MTT assay was repeated with GA-AgNPs_TP-1 and TP-conditioned medium (TCM). Different concentrations (25, 50 and 100 µg/mL) of the treatments were used and the absorbance was determined at 0 min, 5 min, 30 min, 60 min, and 24 h. For the preparation of TCM, GA-AgNPs_TP-1 were diluted in DMEM and were shaken vigorously. Then, the GA-AgNPs_TP-1 were centrifuged for 10 min, and the TCM was collected, filter sterilized, and used for the treatment of BMF cells as described before [48].

### 2.5. Time Dependent Growth Inhibition of the Microbes and the BMF Cells

The aim of this experiment was to determine the time and concentration at which there were less damage to the BMF cells with maximum damage to the microbes. Antimicrobial activity of the GA-AgNPs_TP-1 (25, 50 and 100 µg/mL) was studied by observing microbial growth and counting of the CFU of each sample at different time intervals (0 min, 5 min, 30 min, 60 min and 24 h). Different concentrations of the GA-AgNPs_TP-1 were used starting from 25 to 100 µg/mL. The rate and extent of growth inhibition was determined at each time interval for all the microbes [21]. These effects were compared to the GA-AgNP_TP-1 activity on BMF cells investigated by MTT assay as described in the second experiment of Section 2.4.

### 2.6. Statistical Analysis

All the experiments were carried out in triplicate and the results were analyzed for variance in their respective means using QI Macros 2022 Starter Kit software (KnowWare International, Inc., Denver, CO, USA). The data were presented as means ± SD according to the one-way ANOVA test followed by post hoc, and multiple comparisons (Turkey’s) test. Data were considered statistically significant at *p* value < 0.05.

## 3. Results and Discussion

Oral health plays an important role in the overall health and social aspect of a human being [49]. A number of oral hygiene products are available for the maintenance of oral health, prevention of oral infections, and their progression to oral diseases. Due to the bystander effects of these products and ability of the microbes to develop resistance towards them, new technologies are evolving [41]. The use of AgNPs as alternative antimicrobial, remineralization, and anti-inflammatory agents in dental hygienic products have reached clinical trials [17,19,50]. To date in the NIH U.S. National Library of Medicine ClinicalTrials.gov, only one trial that used green synthesized AgNPs (i.e., thyme and carvacroll based AgNPs) was registered (NCT04431804), and the rest were formulated using chemically-synthesized AgNPs. This suggests that green AgNPs can be used to replace the chemically-synthesized AgNPs and produce biocompatible products for clinical use.

**Scheme 1:**
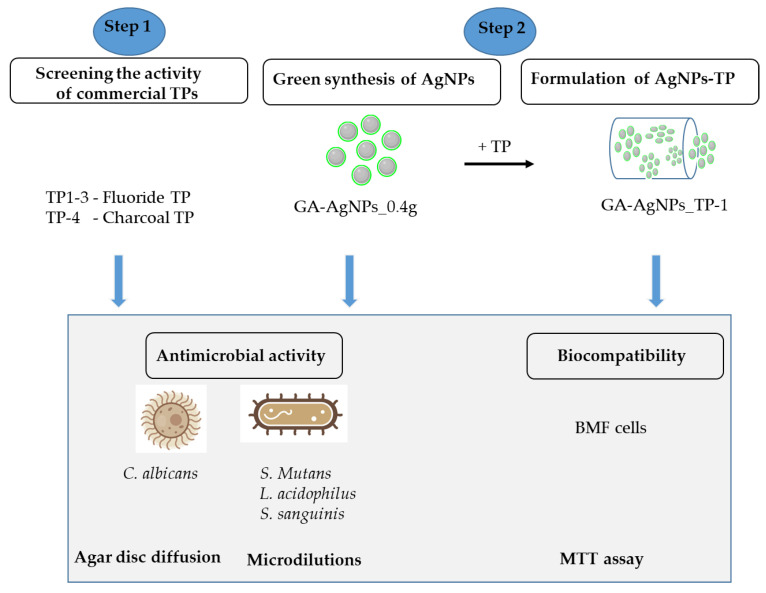
Steps towards GA-AgNPs-TP formulation, investigation of the antimicrobial and cytotoxicity activities. Step 1—screening of commercial TP antimicrobial activity. Step 2—formulation of GA-AgNPs based TP using a TP with least antimicrobial activity.

The current study investigated the potential application of GA-AgNPs as antimicrobial agents in TP formulation; the steps are summarized in Scheme 1. The GA-AgNPs_0.4g were synthesized as previously described and demonstrated antimicrobial activities against Gram positive bacteria, Gram negative bacterial [18,51], and fungal strains. Moreover, these NPs were able to prevent the adhesion of *S*. *mutans* on human tooth enamel, which suggested that they can be used in dentifrices to prevent oral infections [18]. To confirm this claim, four commercial TPs were investigated for their antimicrobial activity on cariogenic and commensal oral microbes to identify the one with the lowest activity. Thereafter, GA-AgNPs_0.4g were incorporated into the selected TP to observe whether its antimicrobial activity was improved.

Various TPs have different efficacy in controlling oral microbes [52,53]. This is influenced by their active ingredients. Differences in the antimicrobial activity of TPs have also been reported in other studies [54]. The selected TPs used in the present study also demonstrated microbial growth inhibition on the test oral microbes. Table 1 shows that all the TPs at 500 µg/mL (stock concentration) showed antimicrobial activity in all microbes, in the following order TP-4>TP-2>TP-3>TP-1. The antimicrobial activities of the TPs were not statistically significant in comparison to each other. However, TP-1 and TP-3 had lower ZOI ranging from 7.00 to 9.41 mm across all the tested microbes compared to the other TPs at 7.77–12.91 mm. Therefore, TP-1 and TP-3 were selected for further studies due to their lowest antimicrobial activity.

**Figure 1:**
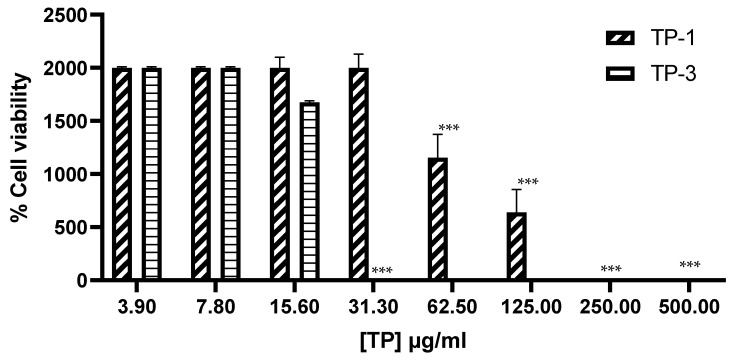
CFU count and MIC of TP-1 and TP-3 on *S*. *mutans*. *p* ≤ 0.05 was considered as statistically significant, *** *p* ≤ 0.001.

In order to select a TP that will be subsequently combined with GA-AgNPs_0.4g, TP-1 and TP-3 were serially diluted (3.9–500 µg/mL) and used in a spot-plating assay to determine the lowest concentration that had no effect on *S. mutans*. The rationale for using *S. mutans* among the four microbes that were studied was that it is the most implicated strain in cariogenesis [55]. TP-1 failed to completely inhibit the growth of *S*. *mutans* but showed a concentration-dependent reduction in CFUs from 13.3 µg/mL as shown Table 2. TP-3 was the most potent and completely inhibited bacterial growth from 62.5 to 500 µg/mL on the spot-plating assay. The resulting CFU counts from TP-1 and TP-3 treatments together with their MICs were further assessed. The observed MICs for TP-1 and TP-3 on *S. mutans* was 125 µg/mL and 31.3 µg/mL for TP-3 (Figure 1). The CFU counts of *S. mutans* exposed to TP-1 and TP-3 demonstrated significant differences in their activities, with TP-1 further being confirmed to have less activity than TP-3. TP-1 treatment kept the *S. mutans* in the lag phase longer than TP-3 treatment. This indicated that TP-3 was more effective in inhibiting *S. mutans* growth than the TP-1. Thus, TP-1 at 31.3 µg/mL was selected for further studies, and used in combination with the GA-AgNPs_0.4g for GA-AgNPs-based TP formulation.

The synthesis and characterization of the GA-AgNPs_0.4g were previously reported; the NPs had a SPR of 425 nm, hydrodynamic size of 220 nm, with a core size range between 4 and 26 nm [18]. The formulation of the TP-1 with GA-AgNPs_0.4g yielded GA-AgNPs_TP-1, which was thereafter tested for antimicrobial activity to determine whether the addition of GA-AgNPs_0.4g to TP-1 will alter its activity. The effect of the GA-AgNPs_TP-1 on all microbes was significantly different (Table 3) compared to TP-1 alone, which had no activity at the test concentration (31.3 µg/mL). Furthermore, the effects of both GA-AgNPs_0.4g and GA-AgNPs_TP-1 on all selected microbes demonstrated a concentration-dependent response, with the highest antimicrobial activity at higher concentrations (50 and 100 µg/mL). The subsequent lower concentrations tested (6.25 and 12.5 µg/mL) demonstrated no activity on all microbes tested.

It must also be noted that GA-AgNPs_0.4g and GA-AgNPs_TP-1 had comparable activities that were not statistically different from each other. This indicated that the activity of GA-AgNPs_0.4g was not affected by adding them to TP-1. AgNPs are highly reactive and might interact with the components of the TP and become inactive. The similarity in the antimicrobial activities of the two treatments suggested that GA-AgNPs can be incorporated in dentifrices and still retain its bioactivity. The GA-AgNPs_0.4g under study showed potential to serve as an antimicrobial agent against the four oral microbes, and prevented adhesion of *S*. *mutans* on human tooth enamels [18]. Several independent studies also highlighted the potential of AgNPs as alternative anti-caries agents when used in the formulation of dentifrices. Nanosilver (TruCareNanosilver) TP, composed of dicalcium phosphate dihydrate, sorbitol, hydrated silica, sodium lauryl sulphate, colloidal silver, menthol, carboxy methyl cellulose, sodium saccharin, flavor, and sodium benzoate compared to fluoride and chitosan TPs was more effective on *S. mutans*. The study indicated that nanosilver TP had the highest antibacterial activity, with ZOI of 20.14 ± 0.96 mm, followed by fluoride TP (16.01 ± 2.68 mm) and chitosan TP (10.84 ± 0.27 mm) [4]. In the literature, there was little evidence on applications of AgNPs as antimicrobial additives in TPs, although there were a significant amount of studies reporting on their enhanced antimicrobial activity using both chemical and plant extract-based AgNPs [9,56]. In a study by Adelere et al., they evaluated the antimicrobial activity of biogenic AgNPs synthesized using an aqueous extract of *Anacardium occidentale* stem bark and incorporated it into a commercial TP. They tested its antibacterial and antifungal activities against *E. coli* and *C. albicans*, respectively, and found that the AgNPs-based TP inhibited the growth of the microbes, while treatments without AgNPs failed to inhibit microbial growth. This demonstrated that the activity was due to the AgNPs added on the TP [22].

The MICs for GA-AgNPs_0.4g and GA-AgNPs_TP-1 were assessed on the four microbes in order to determine the inhibitory concentration that will be non-toxic to dental cells or tissues but still effective in inhibiting bacterial growth. Table 4 shows the response of *S*. *mutans* to the treatments. A similar response was also observed for the other three microbes (data summarized in Table 5). The spot-plating assay revealed a gradient-based reduction effect for the two treatments (GA-AgNPs_0.4g and GA-AgNPs_TP-1) on the selected oral microbes. Microbial growth following exposure to the two treatments was observed at ≤12.5 µg/mL in *S. sanguinis, S. mutans,* and *C. albicans,* and ≤25 µg/mL for *L. acidophilus*. TP-1 had no inhibitory effects on the growth of the microbes at the test concentration.

The MIC_50_ on all microorganisms are presented in Table 5. There were no statistically significant differences between GA-AgNPs_0.4g and GA-AgNPs_TP-1 on individual microbes with reference to their MIC_50_. The MIC_50_ values were slightly higher for GA-AgNPs_TP-1 compared to GA-AgNPs_0.4g. The activity of GA-AgNPs_TP-1 was attributed to the presence of GA-AgNPs_0.4g, as TP-1 was shown in Figure 1 to have no effect on the microbes at the concentration used. The above results suggested that the GA-AgNPs_TP-1 might have promising antimicrobial activity against oral microbes. However, concerns still remained as to whether the GA-AgNPs_TP-1 will have any adverse effects on the oral mucosa. This concern was addressed by testing the effect of GA-AgNPs_TP-1 on BMF cells.

**Figure 2:**
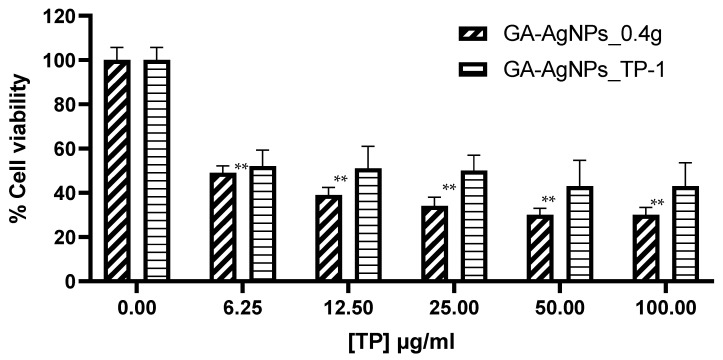
Cytotoxicity effects of GA-AgNPs_0.4g and GA-AgNPs_TP-1 on BMF cells. Cell viability was assessed by MTT assay after treatment for 24 h. *p* ≤ 0.05 was considered as statistically significant, ** *p* ≤ 0.01.

**Figure 3:**
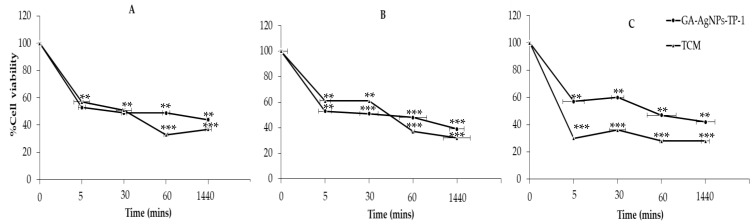
Cytotoxicity effects of GA-AgNPs_TP-1 compared to TCM on BMF cells. Cell viability was assessed by MTT assay after treatment with 25 (**A**), 50 (**B**) and 100 (**C**) µg/mL for various time points up to 24 h. *p* ≤ 0.05 was considered as statistically significant, ** *p* ≤ 0.01 and *** *p* ≤ 0.001.

There was a significant reduction in the number of viable cells after 24 h of treatment with GA-AgNPs_0.4g and GA-AgNPs_TP-1 at all tested concentrations (Figure 2). An interesting finding was that the effect of GA-AgNPs_TP-1 was lower compared to GA-AgNPs_0.4g, suggesting that formulating GA-AgNPs might aid in reducing cytotoxicity of the AgNPs while retaining its antimicrobial activity. This is based on the fact that a reduction in cell viability was also observed with TP-1 alone, which had 72% of viable cells at 31.3 µg/mL. However, it should be noted that the GA-AgNPs_TP-1 had a white deposit which could have influenced the opacity and the results of the assay. Therefore, the TCM was used in parallel with the GA-AgNPs_TP-1 to assess if there were discrepancies with the cytotoxicity assay results. There were no statistically significant differences between the bioactivity of cells treated with GA-AgNPs_TP-1 and TCM at all concentrations tested (Figure 3). Therefore, GA-AgNPs_TP-1 formulation was used for further testing as it had been used for all antimicrobial assays.

**Figure 4:**
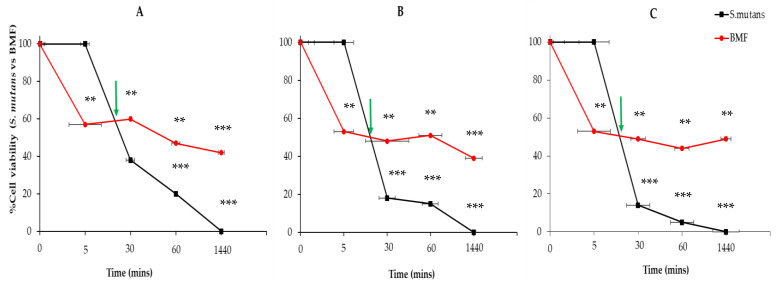
Comparison of the survival of *S. mutans* versus BMF cells after exposure to GA-AgNPs_TP-1. *S. mutans* and BMF cells were treated with 25 (**A**), 50 (**B**) and 100 (**C**) µg/mL for various time points up to 24 h. Viability was evaluated by MTT assay. The green arrow points at an equivalence point of the GA-AgNPs_TP-1. *p* ≤ 0.05 was considered as statistically significant, ** *p* ≤ 0.01 and *** *p* ≤ 0.001.

Due to the cytotoxicity portrayed by the GA-AgNPs_TP-1 on BMF cells, a further cytotoxicity assessment was performed to determine a reference (equivalence) point or the lowest concentration where the treatments show least toxicity to BMF cells but still retain antimicrobial activity. Thus, the doses of GA-AgNPs_0.4g and GA-AgNPs_TP-1 were benchmarked at the lowest concentration that showed antimicrobial activity on the microbes, but had little (~ 80% of viable cells) or no observable adverse effects on the cells. It is desirable that the reference concentration, while active against microorganisms, must be biocompatible and not induce any cellular damage or health risks to humans [23]. In clinical applications, humans are likely to come into contact with the AgNPs through various routes, including oral and skin contact; hence, their biocompatibility must be validated [57]. The analysis of variance for all microbes and BMF cells revealed significant differences, as shown in Figure 4. Treatment with GA-AgNPs_TP-1 demonstrated dose and time dependent effects on the microorganisms (*S. mutans* was used as a representative) and the BMF cells. Both microbial and cell growth declined drastically over this time span. The desired concentration was the one that yielded at least ≥80% BMF cell viability; however, this point occurred much earlier, before 5 min, while all microbes were still at their respective stationary phases. The point of equivalence between the cells and microbes-exposed GA-AgNPs_TP-1 is indicated by an arrow on the graphs (Figure 4), which occurred within the first 30 min for all concentrations of the GA-AgNPs_TP-1; at this point, the cell viability was at 50%. The non-selective effects of GA-AgNPs_0.4g were reported previously [51]: although these effects can be somehow masked in TP formulation (Figure 2), their toxicity still persists. This is quite unexpected since the use of plant-based AgNPs was expected to prevent cytotoxicity associated with chemically-synthesized AgNPs [3,13].

AgNPs toxicity is widely reported in the literature [41,58,59,60], from ingested to cosmetic products, including TPs [16,42]. Their cytotoxicity appears to be time and concentration dependent [43], which was also demonstrated by the current study. Of interest was that the addition of GA-AgNPs_0.4g to TP-1 slightly reduced the cytotoxicity of the GA-AgNPs_0.4g. This effect might be further improved by using other formulations without any additives; of note, TP-1 used in this formulation is a fluoride-based TP and AgNPs are known to be highly reactive and capable of interacting with media components. In this case, it is possible that fluoride can interact with the AgNPs [61]. Nonetheless, the known or common practice of maintaining good oral hygiene is the use of dentifrices for two minutes and rinsing it off [62]. It could therefore be speculated that the GA-AgNPs_TP-1 can be used within the first two minutes of brushing and rinsed without any adverse effects.

## 4. Conclusions

Green synthesis of AgNPs for use in dentifrices is an economical, eco-friendly and promising approach in combating antimicrobial resistance in oral microbes, which will ultimately cause oral diseases. In the current study, GA-AgNPs_0.4g were incorporated into commercial TP at an inactive concentration and demonstrated antimicrobial activity against oral microbes. The antimicrobial activity of GA-AgNPs_0.4g was not altered after being combined with TP-1; the GA-AgNPs_0.4g-TP-1 inhibited the growth of the microbes between 5 min and 24 h of exposure. Although GA-AgNPs_0.4g -TP-1 and GA-AgNPs_0.4g were also shown to be cytotoxic to the oral mucosa-representative (BMF) cells at the same concentrations and the same time intervals that were lethal to the microbes, they can still be used in dentifrices. This is because dentifrices are only used for a short time, are not swallowed, and are rinsed off after a minute of use. Similarly, the GA-AgNPs-based dentifrices can be applied with such cautions. GA-AgNPs_TP-1 has promising prospects for use in dental care to control pathogenic oral microbes, and concerns about its cytotoxicity can be masked or reduced with the addition of biopolymers such as chitosan and poly(ethylene glycol) before its clinical application [63]. Of more interest in this study will be the use of chitosan, which also has antimicrobial activities [4,5]. Therefore, more rigorous studies need to be carried out on oral biofilms and capping off cytotoxicity of GA-AgNPs-based dentifrices. In addition, the phytochemicals involved in the synthesis and capping of the GA-AgNPs_0.4g need to be identified to elucidate the potency, stability, and toxicity of the AgNPs.

## Figures and Tables

**Table 1:** ZOI for commercial TP1-4 against oral microbes.

Microbes	TP-1 (mm)	TP-2 (mm)	TP-3 (mm)	TP-4 (mm)	0.2% CHX (mm)	Nystatin (mm)
*S. sanguinis*	9.32 ± 1.7	10.89 ± 0.76	9.41 ± 0.59	12.91 ± 0.40	17.36 ± 0.31	
*S. mutans*	8.70 ± 0.10	8.55 ± 1.62	8.36 ± 0.12	12.43 ± 0.34	16.22 ± 0.02	
*L. acidophilus*	9.00 ± 0.8	9.8 ± 1.4	9.25 ± 0.08	12.11 ± 0.55	16.99 ± 0.32	
*C. albicans*	7.00 ± 1.44	7.77 ± 3.62	8.19 ± 0.04	11.99 ± 0.35		15.75 ± 0.16

**Table 2:** Effect of TP-1 and TP-3 on *S*. *mutans* using spot-plating assay.

TPs	[TP] µg/mL							
	3.9	7.8	15.6	31.3	62.5	125	250	500
TP-1	+++	+++	+++	+++	++	+	+	-
TP-3	+++	+++	+++	++	-	-	-	-

Note: +, presence of bacterial on the TSA; -, absence of bacterial on the TSA; +++, growth comparable to untreated, ++ and + represent reduction of bacterial growth compared to untreated.

**Table 3:** Antimicrobial activity of GA-AgNPs_0.4g and GA-AgNPs_TP-1 measured as ZOI against selected microbes.

Microbes	[AgNPs] (µg/mL) Added in TP-1	GA-AgNPs_0.4g (mm)	GA-AgNPs_TP-1 (mm)	0.2% CHX (mm)	Nystatin (mm)
*S. sanguinis*	100 50 25	9.11 ± 0.14 8.95 ± 0.32 8.25 ± 0.25	9.17 ± 0.20 9.06 ± 0.08 7.17 ± 0.18	17.29 ± 0.36	
*S. mutans*	100 50 25	9.80 ± 0.19 9.87 ± 0.17 7.19 ± 0.16	9.56 ± 0.39 9.96 ± 0.20 6.14 ± 0.14	16.17 ± 0.16	
*L. acidophilus*	100 50 25	10.04 ± 0.18 9.09 ± 0.09 7.22 ± 0.31	10.23 ± 0.23 9.10 ± 0.09 7.41 ± 0.34	17.00 ± 0.39	
*C. albicans*	100 50 25	10.28 ± 0.18 9.46 ± 0.22 7.12 ± 0.13	10.33 ± 0.20 9.71 ± 0.17 7.31 ± 0.18		15.10 ± 0.15

**Table 4:** Effect of GA-AgNPs_0.4g and GA-AgNPs_TP-1 using spot-plating assay.

TP Formulations	[Formulation] µg/mL				
	6.25	12.5	25	50	100
GA-AgNPs_0.4g	+++	++	-	-	-
GA-AgNPs_TP-1	+++	++	-	-	-

Note: +, presence of bacterial on the TSA; -, absence of bacterial on the TSA; +++, growth com-parable to untreated and TP-1, ++ represent reduction of bacterial growth compared to untreated.

**Table 5:** MIC_50_ for GA-AgNPs_0.4g and GA-AgNPs_TP-1.

Microbes	MIC_50_ (µg/mL)	
	GA-AgNPs_0.4g	GA-AgNPs_TP-1
*S. sanguinis*	10.09	15.66
*S. mutans*	12.26	11.89
*L. acidophilus*	7.12	20.89
*C. albicans*	10.47	12.67

## Data Availability

The data generated in the study are presented as tables and figures in the manuscript.
